# Integration of Proteomics and Metabolomics Into the Design, Build, Test, Learn Cycle to Improve 3-Hydroxypropionic Acid Production in *Aspergillus pseudoterreus*

**DOI:** 10.3389/fbioe.2021.603832

**Published:** 2021-04-07

**Authors:** Kyle R. Pomraning, Ziyu Dai, Nathalie Munoz, Young-Mo Kim, Yuqian Gao, Shuang Deng, Joonhoon Kim, Beth A. Hofstad, Marie S. Swita, Teresa Lemmon, James R. Collett, Ellen A. Panisko, Bobbie-Jo M. Webb-Robertson, Jeremy D. Zucker, Carrie D. Nicora, Henrique De Paoli, Scott E. Baker, Kristin E. Burnum-Johnson, Nathan J. Hillson, Jon K. Magnuson

**Affiliations:** ^1^Pacific Northwest National Laboratory, Richland, WA, United States; ^2^Joint BioEnergy Institute, Emeryville, CA, United States; ^3^Lawrence Berkeley National Laboratory, Berkeley, CA, United States

**Keywords:** 3-hydroxypropionic acid (3-HP), *Aspergillus pseudoterreus*, beta-alanine pathway, Agile BioFoundry, 3HP

## Abstract

Biological engineering of microorganisms to produce value-added chemicals is a promising route to sustainable manufacturing. However, overproduction of metabolic intermediates at high titer, rate, and yield from inexpensive substrates is challenging in non-model systems where limited information is available regarding metabolic flux and its control in production conditions. Integrated multi-omic analyses of engineered strains offers an in-depth look at metabolites and proteins directly involved in growth and production of target and non-target bioproducts. Here we applied multi-omic analyses to overproduction of the polymer precursor 3-hydroxypropionic acid (3HP) in the filamentous fungus *Aspergillus pseudoterreus*. A synthetic pathway consisting of aspartate decarboxylase, beta-alanine pyruvate transaminase, and 3HP dehydrogenase was designed and built for *A. pseudoterreus*. Strains with single- and multi-copy integration events were isolated and multi-omics analysis consisting of intracellular and extracellular metabolomics and targeted and global proteomics was used to interrogate the strains in shake-flask and bioreactor conditions. Production of a variety of co-products (organic acids and glycerol) and oxidative degradation of 3HP were identified as metabolic pathways competing with 3HP production. Intracellular accumulation of nitrogen as 2,4-diaminobutanoate was identified as an off-target nitrogen sink that may also limit flux through the engineered 3HP pathway. Elimination of the high-expression oxidative 3HP degradation pathway by deletion of a putative malonate semialdehyde dehydrogenase improved the yield of 3HP by 3.4 × after 10 days in shake-flask culture. This is the first report of 3HP production in a filamentous fungus amenable to industrial scale biomanufacturing of organic acids at high titer and low pH.

## Introduction

Since the industrial revolution, petroleum-based feedstocks have been the primary source for production of fuels and chemicals. However, their non-renewable nature and the detrimental effects of extractive practices have fueled a movement to produce drop-in or alternative fuels and chemicals from renewable feedstocks. A bioeconomy, based around the production of fuels and chemicals from renewable biological feedstocks has been proposed and supported as a way to improve the sustainability of fuel and commodity chemical production. Building new and modifying existing industries to produce the myriad of chemicals currently derived from petrochemical feedstocks will require extensive metabolic engineering of a wide variety of bacteria, plants, and fungi and integration with catalytic and thermochemical conversion processes to attain near-term economic viability. To support this, a public Agile BioFoundry has been established to efficiently engineer microorganisms for the production of fuels and chemicals from renewable feedstocks^1^ (Hillson et al., 2019). The breadth of capabilities available to Design, Build, Test, and Learn (DBTL) from engineered organisms at a dedicated biofoundry enables a system-wide examination of engineered pathways and a deeper understanding of metabolic capability in non-model bioconversion hosts. An emphasis on maximizing the efficiency of multi-omic analyses during the Test and Learn portions of the DBTL cycle will expedite the arrival of a functioning bioeconomy by allowing strain and bioprocess development to overcome challenges associated with the complexity of metabolic systems.

Of interest to supporting a sustainable bioeconomy are biological production of chemicals that can be polymerized to produce biobased and biodegradable plastics. One such chemical, 3-hydroxypropionic acid (3HP) can be used to produce polymers directedly, such as poly-3HP, or more complex structures that also incorporate 3-hydroxybutyric acid, 4-hydroxybutyric acid, and/or lactic acid to produce polymers with altered physical properties (Tripathi et al., 2013; Meng et al., 2015; Ren et al., 2017). For drop-in to existing industrial processes, 3HP can be dehydrated to produce acrylic acid (Decoster et al., 2013) which has received commercial interest for biobased production of acrylates from glycerol (BASF, 2014). 3HP is also a precursor for carbon fiber via conversion to acrylonitrile and polymerization to polyacrylonitrile, which can be used in standard industrial processes to make carbon fibers (Davey, 2018).

3HP is produced naturally by phototrophic bacteria in the family *Chloroflexaceae* capable of CO_2_ fixation via the 3HP cycle (Strauss and Fuchs, 1993; Klatt et al., 2007) as well as chemotrophic *Crenarchaeota* via the 3HP/4HB cycle (Berg et al., 2007). Enzymes from naturally occurring 3HP biosynthetic pathways have been used to design 3HP production pathways that route metabolic flux through beta-alanine, malonyl-CoA, or glycerol (Valdehuesa et al., 2013; Ji et al., 2018). Iterations of these pathways have been engineered into a variety of yeast and bacteria with notable successes. In the yeast *Saccharomyces cerevisiae* both the malonyl-CoA (Kildegaard et al., 2016) and beta-alanine (Borodina et al., 2015) pathways have been established with yields of 0.13 and 0.14 g/g 3HP from glucose. In bacteria production of 3HP via the glycerol pathway has been particularly successful with yields as high as 0.457 g/g 3HP directly from glycerol with engineered *Escherichia coli* (Kim et al., 2020) and 0.51 g/g 3HP from glucose with engineered *Corynebacterium glutamicum* which is also capable of converting xylose to 3HP (Chen et al., 2017). These examples demonstrate that microorganisms can be engineered to produce 3HP at reasonably high levels in laboratory conditions and further research will continue to improve production efficiency toward levels needed for economic viability. However, apart from *C. glutamicum*, the organisms being engineered for 3HP production may not be ideal hosts for bioconversion of lignocellulosic and low-cost renewable feedstocks that are often composed of a spectrum of organic compounds that model organisms may struggle to metabolize. Further, production of 3HP as a free acid, rather than its conjugate base may aid online or down-stream separation processes. Filamentous fungi from the genus *Aspergillus* are notable for their ability to produce organic acids such as citric acid, malic acid, and itaconic acid at industrial scale and at a pH that is well below the pKa = 4.5 of 3HP (Liu et al., 2017; Cairns et al., 2018; Kuenz and Krull, 2018). To that end, we examined utilization of *Aspergillus pseudoterreus* as a host for bioconversion of sugars to 3HP through the beta-alanine pathway (Borodina et al., 2015) because of its acid tolerance, high glycolytic flux, and ability to utilize a broad range of carbon and nitrogen sources (Kuenz and Krull, 2018; Saha et al., 2019; Deng et al., 2020). Engineered strains were evaluated using multi-omics analyses to understand the impacts of the introduced metabolic pathway and identify targets for improvement of 3HP production.

## Materials and Methods

### Strain Maintenance and Cultivation

*Aspergillus pseudoterreus* strain ATCC^®^ 32359^TM^ was obtained from American Type Culture Collection (Manassas, VA, United States). All strains were maintained on complete medium agar (CM) (Deng et al., 2020) and spore suspensions stored in 15% glycerol at –80°C. Spore inoculum was grown on CM agar plates for 5 days to produce conidia, which were harvested by washing the plates with sterile 0.4% Tween 80 solution. For shake-flask experiments, 2E6 *A. pseudoterreus* spores were inoculated in 50 mL minimal medium [MM; 100 g/L glucose, 2.36 g/L (NH_4_)_2_SO_4_, 0.11 g/L KH_2_PO_4_, 2.08 g/L MgSO_4_.7H_2_O, 0.13 g/L CaCl_2_.2H_2_O, 74 mg/L NaCl, 1.3 mg/L ZnSO_4_.7H_2_O, 0.7 mg/L MnCL_2_.4H_2_O, 5.5 mg/L FeSO_4_.7H_2_O, 0.2 mg/L CuSO_4_.5H_2_O, adjusted to pH 3.4 with 5M KOH] adapted from Riscaldati et al. (2000) in 250 mL shake-flasks at 30°C and 200 rpm in an orbital shaker. For submerged stirred tank 20 L bioreactor cultivation, 2E6 *A. pseudoterreus* spores were inoculated within a 30-L Sartorius Biostat-C bioreactor filled to 20 L with MM at 30°C. Impeller stir-rate was set at a constant rate of 100 rpm until the pH dropped to 2.8 after which stir-rate was increased to 400 rpm and pH maintained at 2.8 by online addition of 5M KOH. For submerged stirred tank 0.5 L bioreactor cultivation, 2E6 *A. pseudoterreus* spores were inoculated within 0.5 L Sixfors bioreactors (Infors HT, Basel, Switzerland) filled to 0.45 L with MM at 30°C. Impeller stir-rate was set at a constant rate of 200 rpm until the pH dropped to 2.8 after which stir-rate was increased to 600 rpm and pH maintained at 2.8 by online addition of 5M KOH. For both reactor scales air was sparged at a constant rate of 0.4 vessel volumes/min. Sterile broth samples were collected by filtration through a 0.2 μm filter and then frozen at –20°C for analysis of sugar, nutrient, and metabolite concentrations. Cell pellets were collected by vacuum filtration on 0.45 μm nylon Whatman filters and washed with phosphate buffered saline. Cells were transferred to Eppendorf tubes, flash frozen in liquid nitrogen, and stored at –80°C. Mycelial dry cell weight at each time point was determined by harvesting the mycelia on a pre-weighed filter by vacuum filtration and washing with 20 mL distilled water. Subsequently, the dry weight was determined after freeze-drying in a lyophilizer overnight.

### Strain Construction

Coding sequences for *Tc*PAND, *Bc*BAPAT, and *Ec*HPDH were codon optimized for *Aspergillus* species and synthesized. Individual DNA fragments were isolated with the following primer pairs; 5′-*cad1* of *A. pseudoterreus* 5′-(ccctcgaggtcgacggtat cgatagatatcggttgtagcagcgtaaacac-3′/5′-tctttcatagtagccttggtgaacatc ttgagg-3′); *gpdA* promoter of *A. niger* (5′-atgttcaccaaggctac tatgaaagaccgcgatg-3′/5′-cgccggtggcgggcattgtttagatgtgtctatgtg-3′); *Tc*PAND coding sequence (5′-catctaaacaatgcccgccaccggcgagga cca-3′/5′-atccaacccatcagaggtcggagcccaggcgttcg-3′); bi-direction transcriptional terminator of *A. niger elf3* gene (5′-gggct ccgacctctgatgggttggatgacgatg-3′/5′-tctggcccagctctgagtcctagatgggt ggtg-3′); *Bc*BAPAT coding sequence (5′-catctaggactcagagctg ggccagacattccttc-3′/5′-gtccatcaacatggaactgatgatcgtccaggtcac-3′); and *A. niger eno1* promoter (5′-cgatcatcagttccatgttgatggactgga ggg-3′/5′-gaactagtggatcccccgggctgcgttaactcgagcttacaagaagtagcc-3′) by high-fidelity PCR and assembled by Gibson Assembly (Gibson et al., 2008) into the pBlueScript SK(–) vector linearized with restriction enzymes *Hin*dIII and *Pst*I to form pZD4025 with an addition of *Hpa*I restriction enzyme site downstream of the *enol* promoter.

Individual DNA fragments were then isolated with the following primer pairs; *gpdA* (or *tdh*) promoter of *A. nidulans* (5′-acaggctacttcttgtaagctcgagtttctgtacagtgaccggtgac-3′/5′-tgaccag cacgatcatggtgatgtctgctcaag-3′); EcHPDH coding sequence (5′-agacatcaccatgatcgtgctggtcacgggcgc-3′/5′-gccatcggtcctattggcg gtggacgttcaggc); *trpC* transcriptional terminator (5′-cgtccaccgcca ataggaccgatggctgtgtag-3′/5′-cccgtctgtcagagagcggattcctcagtctcg-3′); pyrithiamine resistance gene (ptrA) of *A. oryzae* (5′-gaggaatccgctctctgacagacgggcaattgattac-3′/5′-gaatgttgctgagga gccgctcttgcatctttg-3′); 3′-*cad1* gene (5′-gcaagagcggctcctcagcaac attcgccatgttc-3′/5′-actaaagggaacaaaagctggagctcagctccactgctcata gtctttg-3′) by PCR and assembled along with *Hpa*I linearized pZD4025 to form pZD4028. pZD4028 was linearized with *Eco*RV for chemical mediated protoplast transformation into *A. pseudoterreus* (ATCC^®^ 32359^TM^) as previously described (Deng et al., 2020) to construct strains ABF_004528_2 (*cad*:3HP+) and ABF_004528_6 (*cad*:3HP+, 3HP+).

Upstream and downstream regions of the candidate malonate semialdehyde dehydrogenase (Ap*ald6*) were PCR amplified from *A. pseudoterreus* genomic DNA using primer pairs (5′-gcctataagcaaccgcctgtataaggaagagccttggtgctaacggc-3′/5′-gctg cgcaactgttgggaagggcgatgctgccagactgcaacaagaacc-3′) and (5′-g cagcccagtagtaggttgaggccgtcgacgaagctgatggccttgatgg-3′/5′-tgttgac ctccactagctccagccaaggcttctcgcactctcaccaccgc-3′) respectively. Hygromycin phosphotransferase (*hph*) was amplified with oligo pair (5′-cttggctggagctagtggagg-3′/5′-tccttatacaggcggttgc-3′) and the backbone of vector pRF-HU2 (Frandsen et al., 2008) with oligo pair (5′-atcgcccttcccaacagttgcg-3′/5′-acggc ctcaacctactactgggc-3′). The four fragments were assembled using NEBuilder^®^ HiFi DNA Assembly Master Mix (NEB, Ipswitch, MA, United States) according to the manufacturer’s instructions and transformed into strain ABF_004528_6 (*cad*:3HP+, 3HP+) using agrobacterium mediated transformation as previously described (Michielse et al., 2008) to make strain ABF_008339 (*cad*:3HP+, 3HP+; ΔAp*ald6*).

### Sample Preparation

TissueLyser II system (Qiagen, Valencia, CA, United States) 2 mL trays were frozen at –20°C overnight. Two 3 mm stainless steel beads were added to 2 mL snap-cap centrifuge tubes (Eppendorf, Hamburg, Germany) along with 0.5 mL H_2_O and each fungus piece. The tubes were placed inside the trays and they were then set on the TissueLyser II system. The frozen samples were ground for 2 min at 30 Hz until it reached a completely homogenized, cold solution. To separate the protein and metabolites, 1 mL cold (–20°C) chloroform:methanol mix [prepared 2:1 (v/v)] was pipetted into a chloroform compatible 2 mL Sorenson MulTI^TM^ SafeSeal^TM^ microcentrifuge tubes (Sorenson bioscience, Salt Lake City, UT, United States) inside an ice-block. Then 200 μl of the sample homogenate in water was added to the Sorenson tube at a ratio of 1:5 sample:chloroform mix [2:1 (v/v)] and vigorously vortexed. The sample was then placed in the ice block for 5 min and then vortexed for 10 s followed by centrifugation at 10,000 × *g* for 10 min at 4°C. The upper water-soluble metabolite phase and the lower lipid soluble phase were also collected into the same vial for metabolomics analysis. The metabolite samples were dried to complete dryness in a speed vac and then capped and stored dry at –20°C until analysis. The remaining protein interphase had 1 mL of cold (–20°C) pure methanol added, vortexed and centrifuged at 12,000 ×*g* for 5 min to remove the chloroform and pellet the protein. The methanol supernatant was decanted into waste and the pellet lightly dried in a fume hood and then stored at –80°C until protein digestion.

### Protein Digestion

The protein interlayer pellet was digested by adding 200 μl of an 8 nM urea solution to the protein pellets and vortexed into solution. A bicinchoninic acid (BCA) assay (Thermo Scientific, Waltham, MA, United States) was performed to determine protein concentration. Following the assay, 10 mM dithiothreitol (DTT) was added to the samples and incubated at 60°C for 30 min with constant shaking at 800 rpm. Reduced cysteine residues were alkylated by adding 400 mM iodoacetamide (Sigma-Aldrich) to a final concentration of 40 mM and incubating in the dark at room temperature for 1 h. Samples were then diluted eightfold for preparation for digestion with 100 mM NH_4_HCO_3_, 1 mM CaCl_2_ and sequencing-grade modified porcine trypsin (Promega, Madison, WI, United States) was added to all protein samples at a 1:50 (w/w) trypsin-to-protein ratio for 3 h at 37°C. Digested samples were desalted using a 4-probe positive pressure Gilson GX-274 ASPEC^TM^ system (Gilson Inc., Middleton, WI, United States) with Discovery C18 100 mg/1 mL solid phase extraction tubes (Supelco, St. Louis, MO, United States), using the following protocol: 3 mL of methanol was added for conditioning followed by 2 mL of 0.1% triflouroacetic acid (TFA) in H_2_O. The samples were then loaded onto each column followed by 4 mL of 95:5: H_2_O:ACN, 0.1% TFA. Samples were eluted with 1 mL 80:20 ACN:H_2_O, 0.1% TFA. The samples were concentrated down to ∼100 μL using a Speed Vac and a final BCA was performed to determine the peptide concentration, and then stored at –80°C until usage.

### Global Proteomics Analysis

Peptides digests were diluted to 0.1 μg/μL with nanopure water for LC-MS/MS analysis. Five μL of samples were loaded onto in-house packed reversed-phase capillary columns (70 cm × 75 μm i.d.) with 3 μm Jupiter C18 particles. The separation was carried out using a nanoAcquity HPLC system (Waters Corporation, Milford, MA, United States) at room temperature. The mobile phase A is 0.1% formic acid in water while the mobile phase B is 0.1% formic acid in acetonitrile. The elution was carried out at 300 nL/min with the following gradient: 0–2 min 1% B; 2–20 min 8% B; 20–75 min 12%B; 75–97 min 30%B; 97–100 min 45%; 100–105 95%; 105–110 min 95%; 110–140 min 1%. The eluting peptides were directly analyzed using a Q Exactive HF mass spectrometer (Thermo Fisher Scientific) in data-dependent acquisition mode. Mass spectrometer settings were as following: full MS (AGC, 3 × 10^6^; resolution, 60,000; m/z range, 300–1800; maximum ion time, 20 ms); MS/MS (AGC, 1 × 10^5^; resolution, 15000; m/z range, 200–2000; maximum ion time, 100 ms; TopN, 12; isolation width, 2 Da; dynamic exclusion, 30.0 s; collision energy, NCE 30).

All mass spectrometry data were searched using MS-GF+ (Kim and Pevzner, 2014) and MASIC (Monroe et al., 2008) software. MS-GF + software was used to identify peptides by scoring MS/MS spectra against peptides derived from the whole protein sequence database. MASIC software was used to generate the selected ion chromatographs (SICs) of all the precursors in MSMS datasets and calculate their peak areas as abundance. MASICResultsMerger^2^ was used to append the relevant MASIC stats for each peptide hit result in MS-GF+. The MS-GF+ data were then filtered based on 1% false discovery rate (FDR) and less than 5-ppm mass accuracy to generate a list of qualified peptide-hit results. The abundance of peptides was determined as the highest peak area identified for the peptide within a sample.

Sample level quality was ensured by a robust Principal Component Analysis (PCA) to compute a robust Mahalanobis distance (rMd) based on sample-level parameters (Matzke et al., 2011). Peptides were also filtered to remove those with inadequate data for statistics, either three samples with measured values in one group or two values measured for subjects within two distinct groups. The default for normalization is standard global median centering to account for total abundance differences between samples. A test was performed to assure that these factors are not biases (Webb-Robertson et al., 2010). For this dataset there was no bias detected and we utilized global median centering (Callister et al., 2006). Protein quantification was performed with standard reference-based median averages (Polpitiya et al., 2008; Matzke et al., 2013). Statistics were performed with established standard methods (Webb-Robertson et al., 2017). For time we utilized an ANOVA with a Dunnett’s test to compare all time points back to the first time point within each strain. We also utilized a g-test with a Bonferonni correction to identify qualitative markers both to compare strains or time (Webb-Robertson et al., 2010).

### Targeted Proteomics Analysis

Targeted Proteomics was performed via Liquid Chromatography (LC) – Selected Reaction Monitoring (SRM) approach. 3–5 peptides per protein were initially selected based on either their identification in global proteomics or their SRM suitability scores predicated by CONSeQuence (Eyers et al., 2011) and Prego (Searle et al., 2015) software tools. All the peptides were further blasted to ensure their uniqueness to target proteins in the organism. Crude synthetic heavy isotope-labeled (e.g., 13C/15N on C-terminal lysine and arginine) peptides were purchased from New England Peptide (Gardner, MA, United States). Upon receiving, the crude synthetic heavy peptides were mixed together and diluted with 0.1% formic acid in 15% acetonitrile in water to obtain a nominal concentration of 1.5 pmol/μL for each individual peptide. The heavy peptide mixture stock solution was aliquoted and stored at –80°C until further use.

To develop targeted proteomics assay, all the SRM precursor-fragment ion pairs (i.e., transitions) were first analyzed using LC-SRM by spiking heavy peptides in test samples. 2–3 transitions per peptide and 1–5 peptides per protein were selected in a final assay based on their LC performance, MS response, transition interferences, and endogenous peptide detectability. Collision energies of transitions were obtained using empirical equations provided in Skyline software (MacLean et al., 2010).

For peptide samples, crude heavy peptide mixture stock solution was spiked in the 0.25 μg/μL peptide samples at a nominal concentration of 35 fmol/μL for each peptide. LC-SRM analysis utilized a nanoACQUITY UPLC^®^ system (Waters Corporation, Milford, MA, United States) coupled online to a TSQ Altis^TM^ triple quadrupole mass spectrometer (Thermo Fisher Scientific). The UPLC^®^ system was equipped with an ACQUITY UPLC BEH 1.7 μm C18 column (100 μm i.d. × 10 cm) and the mobile phases were (A) 0.1% formic acid in water and (B) 0.1% formic acid in acetonitrile. Two μL of sample (i.e., 0.4 μg of peptides) were loaded onto the column and separated using a 110-min gradient profile as follows (min:flow-rate-μL/min:%B): 0:0.4:1, 6:0.6:1, 7:0.4:1, 9:0.4:6, 40:0.4:13, 70:0.4:22, 80:0.4:40, 85:0.4:95, 91:0.5:95, 92:0.5:95, 93:0.5:50, 94:0.5:95, 95:0.6:1, 98:0.4:1. The LC column is operated with a temperature of 42°C. The TSQ Altis^TM^ triple quadrupole mass spectrometer was operated with ion spray voltages of 2100 ± 100 V and a capillary inlet temperature of 350°C. Tube lens voltages were obtained from automatic tuning and calibration without further optimization. Both Q1 and Q3 were set at unit resolution of 0.7 FWHM and Q2 gas pressure was optimized at 1.5 mTorr. The transitions were scanned with a 7 min retention time window and a cycle time of 0.8 s.

All the LC-SRM data were imported into Skyline software and the peak boundaries were manually inspected to ensure correct peak assignment and peak boundaries. Peak detection and integration were determined based on two criteria: (1) the same LC retention time and (2) approximately the same relative peak intensity ratios across multiple transitions between the light peptides and heavy peptide standards. The total peak area ratios of endogenous light peptides and their corresponding heavy isotope-labeled internal standards were then exported from Skyline software as Ratio-to-Standard. For each peptide, the total peak area ratios of individual samples were normalized to the average total peak area ratio of all the samples.

Sample level quality was ensured by a robust Principal Component Analysis (PCA) to compute a robust Mahalanobis distance (rMd) based on sample-level parameters (Matzke et al., 2011). The default for normalization is standard global median centering to account for total abundance differences between samples. A test was performed to assure that these factors are not biases (Webb-Robertson et al., 2010). For this dataset, we utilized global median centering (Callister et al., 2006). Protein quantification was performed with standard reference-based median averages (Polpitiya et al., 2008; Matzke et al., 2013). Statistics included standard methods (Webb-Robertson et al., 2017). For this specific dataset, we utilized an analysis of variance (ANOVA) with Tukey’s adjustment to compare all strains to on another within each time point. In the time-course study, we utilized an ANOVA with a Dunnett’s test to compare all time points back to the first time point within each strain.

### Global Intracellular Metabolomics

The stored metabolite extracts were completely dried under speed-vacuum to remove moisture and chemically derivatized as previously reported (Kim et al., 2015). Briefly, the extracted metabolites were derivatized by methoxyamination and trimethylsilyation (TMS), then the samples were analyzed by GC-MS. Samples were run in an Agilent GC 7890A using a HP-5MS column (30 m × 0.25 mm × 0.25 μm; Agilent Technologies, Santa Clara, CA, United States) coupled with a single quadrupole MSD 5975C (Agilent Technologies). One microliter of sample was injected into a splitless port at constant temperature of 250°C. The GC temperature gradient started at 60°C and hold for 1 min after injection, followed by increase to 325°C at a rate of 10°C/min and a 5-min hold at this temperature. Fatty acid methyl ester standard mix (C8-28) (Sigma-Aldrich) was analyzed in parallel as standard for retention time calibration. GC-MS raw data files were processed using the Metabolite Detector software (Hiller et al., 2009). Retention indices (RI) of detected metabolites were calculated based on the analysis of a FAMEs mixture, followed by their chromatographic alignment across all analyses after deconvolution. Metabolites were initially identified by matching experimental spectra to a PNNL augmented version of Agilent GC-MS metabolomics Library, containing spectra and validated retention indices for over 850 metabolites. Then, the unknown peaks were additionally matched with the NIST17/Wiley11 GC-MS library. All metabolite identifications and quantification ions were validated and confirmed to reduce deconvolution errors during automated data-processing and to eliminate false identifications.

### Quantification of Extracellular Metabolites

For quantification of metabolites in the spent medium, samples were diluted 1:20 and 20 μL of these diluted samples were dried in a glass vial. Solutions ranging from 0 to 300 μg/mL containing standards of organic acids of interest, including 3HP, were prepared and 20 μL of these in triplicate were dried to build an external calibration curve. Derivatization protocol, analysis by GC-MS and processing of the data was done as described above, but the analysis was targeted to only the metabolites present in the standard mixes. Calibration curves were established for each of the standards in the mixture of organic acids. After linear regression fitting, concentration of the metabolites in the spent medium samples was determined upon consideration of the dilution factor. For HPLC, samples were analyzed using high performance liquid chromatography (HPLC) equipped with a Waters 2414 refractive index detector. A Bio-Rad Aminex HPX-87H ion exclusion column (300 mm × 7.8 mm), heated to 65°C was used for analyte separation. Sulfuric acid (0.0045 M) was used as eluent at a flow rate of 0.55 mL/min. Extracellular metabolite quantities were used as boundary constraints for parsimonious flux balance analysis (pFBA) using OptFlux (Vilaca et al., 2018) with *Aspergillus niger* genome scale metabolic model iJB1325 (Brandl et al., 2018).

## Results

### Design and Build to Establish 3HP Production in *Aspergillus pseudoterreus*

**FIGURE 1:**
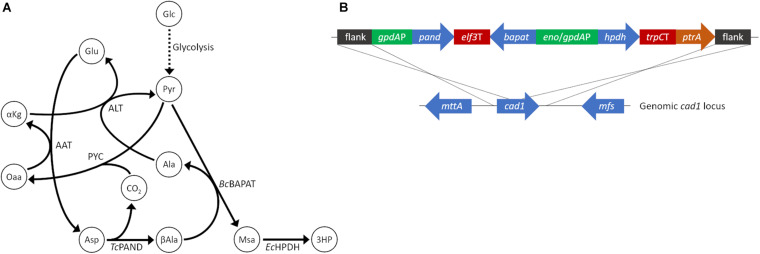
Establishment of 3HP production in *A. pseudoterreus*. **(A)** Ideal carbon flux in a high theoretical yield (2 mol/mol) metabolic pathway for production of 3HP from sugar monomers. **(B)** The beta-alanine metabolic pathway to enable 3HP production was codon optimized for *A. pseudoterreus* and placed under the control of high expression promoters. The engineered pathway was targeted to the *cis*-aconitic acid decarboxylase (*cad*1) locus to disrupt the gene. 3HP, 3-hydroxypropionate; AAT, aspartate aminotransferase, Ala, alanine; ALT, alanine transaminase; αKg, alpha-ketoglutarate; Asp, Aspartate; βAla, beta-alanine; *Bc*BAPAT, beta-alanine pyruvate aminotransferase; *Ec*HPDH, 3-hydroxypropionate dehydrogenase; Glc, glucose; Glu, glutamate; Msa, malonate semialdehyde; Oaa, oxaloacetate; PYC, pyruvate carboxylase; Pyr, pyruvate; *Tc*PAND, aspartate 1-decarboxylase.

**FIGURE 2:**
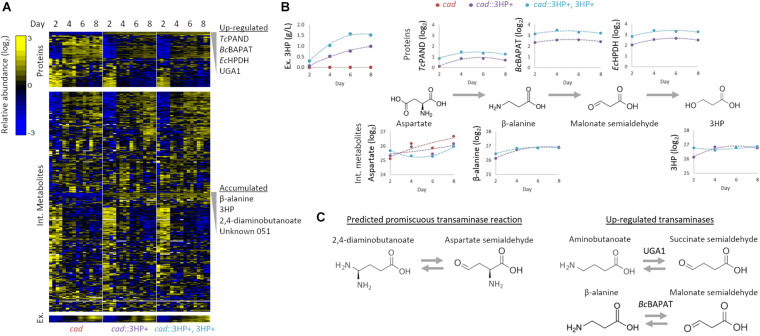
Multi-omics analysis of 3HP producing *A. pseudoterreus* strains. **(A)** Profiling of intracellular and extracellular metabolites and targeted proteomics of central metabolic pathways. **(B)** Effect of establishment and increase in production level on enzymes and metabolites in the engineered 3HP metabolic pathway. **(C)** The metabolite 2,4-diaminobutanoate is accumulated in engineered strains. Upregulated transaminases that may promiscuously use aspartate semialdehyde as a substrate are shown.

Production of 3HP in fungi has been explored in a number of species including *Pichia pastoris* (Zhou et al., 2011), *Schizosaccharomyces pombe* (Takayama et al., 2018), and via a variety of pathways in *S. cerevisiae* (Borodina et al., 2015; Kildegaard et al., 2016). However, to our knowledge 3HP production has not been explored in filamentous fungi. *Aspergillus* species, in particular, are notable for their ability to produce commodity chemicals at low pH which is valuable for the production of free acids. We chose *A. pseudoterreus* as a filamentous fungal host in which to establish 3HP production due to its demonstrated ability to produce and tolerate high quantities of itaconic acid at low pH (Okabe et al., 2009). The beta-alanine production pathway (Figure 1A), originally demonstrated in *S. cerevisiae* (Borodina et al., 2015), was recapitulated for *A. pseudoterreus* by codon optimizing the aspartate-1-decarboxylase (*pand*), beta-alanine pyruvate transaminase (*bapat*) and 3-hydroxypropionate dehydrogenase (*hpdh*) genes from *Tribolium castaneum*, *Bacillus cereus*, and *E. coli* respectively; and expressing them under the control of high-expression *gpdA* and *enoA* promoters (Figure 1B). The major product secreted by *A. pseudoterreus* during industrial fermentations is itaconic acid (Lockwood and Nelson, 1946). To eliminate production of itaconic acid, the beta-alanine pathway overexpression construct was targeted to the *cis*-aconitic acid decarboxylase (*cad1*) locus (Deng et al., 2020) to allow redistribution of metabolic flux elsewhere (Figure 1B). Screening of transformants identified a transgenic strain with a single copy of the synthetic pathway integrated at the *cad1* locus (strain ABF_004528_2; *cad*:3HP+) and a strain with one copy of the synthetic pathway integrated at the *cad1* locus and a second copy of the pathway that integrated elsewhere in the genome (strain ABF_004528_6; *cad*:3HP+, 3HP+). Deletion of *cad1* (strain ABF_002234) eliminated itaconic acid production as previously described (Deng et al., 2020). Overexpression of the beta-alanine pathway established 3HP production in *A. pseudoterreu*s while a second copy of the biosynthetic pathway enabled nearly twice the yield of extracellular 3HP (Figure 2B).

### Test/Learn; Multi-Omics Analysis of 3HP

3HP producing strains with single and multi-copy integration of the beta-alanine 3HP pathway were selected and compared to a *cad1* parental strain during an 8-day production period in shake flasks. Flasks were harvested at 2-day intervals and samples collected for intracellular and extracellular metabolomics as well as global and targeted proteomics. In total, global profiling detected 4,674 proteins, while targeted proteomics quantified 79 proteins involved in central carbon metabolism. Nine extracellular metabolites were absolutely quantified while 320 intracellular metabolites were detected, 122 of which were structurally identified (Figure 2).

Individual metabolites and proteins were assessed to identify metabolic nodes and pathways that are perturbed in *Aspergillus* strains engineered for 3HP production. The most strongly perturbed proteins and metabolites identified are directly involved in 3HP production as intermediates to the biosynthetic pathway. In the strain with the synthetic 3HP pathway integrated at multiple locations in the genome, we detected 1.66 ± 0.13× as much of the three heterologous proteins (*Tc*PAND, *Bc*BAPAT, and *Ec*HPDH) (Figure 2B). Conversely, the intracellular levels of beta-alanine and 3HP are not significantly different in the engineered strains suggesting that the production of extracellular 3HP is not transport limited, nor is the flux toward 3HP limited by either of these reactions. The fact that aspartate is present at a somewhat higher level in the single-copy strain suggests that flux through Aspartate 1-decarboxylase may be the limiting step in the pathway.

The engineered 3HP biosynthetic route from pyruvate involves a series of balanced transamination reactions (alanine transaminase, aspartate aminotransferase, and the introduced beta-alanine pyruvate transaminase; Figure 1A) that under ideal conditions cycle nitrogen between alanine, glutamate, aspartate, and beta-alanine. Establishment and increase in expression level of the 3HP biosynthetic pathway did not significantly alter the alanine, glutamate, or aspartate pool sizes though they did tend to decrease in concentration. The beta-alanine pool size substantially increased (148 × – 249 × higher from day 2 to 8) with establishment of the pathway but was not significantly altered by higher expression level. In addition to beta-alanine, we found that the pool size of 2,4-diaminobutanoate is substantially increased (41 × –131 × higher from day 2 to 8) with establishment of the pathway. Metabolic pathways that produce 2,4-diaminobutanoate are not annotated in *Aspergillus* species. We therefore examined the proteome for up-regulated transaminases capable of producing 2,4-diaminobutanoate from substrates that are enriched or suspected to be high-flux in the engineered strains. The most significantly up-regulated enzyme in the engineered strains is the 4-aminobutyrate aminotransferase Ap*uga1*. If Ap*uga1*also functions promiscuously with aspartate-semialdehyde as a substrate (Figure 2C), 2,4-diaminobutanoate would be produced rather than 4-aminobutanoate (predicted reaction: aspartate-semialdehyde + glutamate ↔ alpha-ketoglutarate + 2,4-diaminobutanoate). It is also possible that *Bc*BAPAT, overexpressed as part of the engineered 3HP pathway, is promiscuous and produces 2,4-diaminobutanoate with aspartate-semialdehyde as a substrate (predicted reaction: aspartate-semialdehyde + alanine ↔ pyruvate + 2,4-diaminobutanoate). We did not detect 2,4-diaminobutanoate extracellularly suggesting that this by-product of the engineered pathway accumulates as an intracellular nitrogen sink and may compete with nitrogen recycling necessary for high-yield 3HP production.

### Test/Learn; 3HP Scale-Up

**FIGURE 3:**
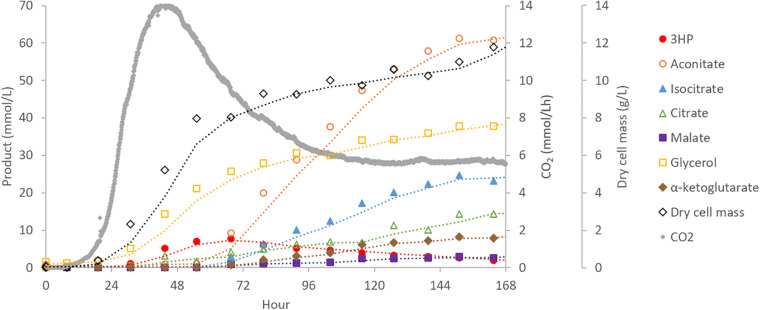
Bioreactor cultivation of *A. pseudoterreus* for 3HP production. Time-course production of 3HP and other bioproducts detected by extracellular metabolomics.

To assess performance changes during scale-up, the highest producing 3HP strain from shake-flask experiments (*cad*:3HP+, 3HP+) was cultured in a 30 L bioreactor. Off-gas, biomass, and targeted extracellular metabolite concentrations were monitored to provide boundary constraints for parsimonious flux balance analysis (pFBA) (Vilaca et al., 2018). During bioreactor cultivation, biomass and CO_2_ were the main products during the first 2 days of cultivation with CO_2_ production peaking at hour 44. Production of 3HP and glycerol began early during the exponential growth phase of the culture and prior to peak CO_2_ production. In contrast, production of organic acids (aconitic acid, isocitric acid, citric acid, alpha-ketoglutaric acid, and malic acid) began later when biomass production had slowed and become a near-linear process (Figure 3). This suggests that production of 3HP via the beta-alanine pathway is primarily a growth linked process, and as such, scale-up requirements may be very different than bioprocesses optimized for production of TCA-cycle derived organic acids in *Aspergillus* species.

**FIGURE 4:**
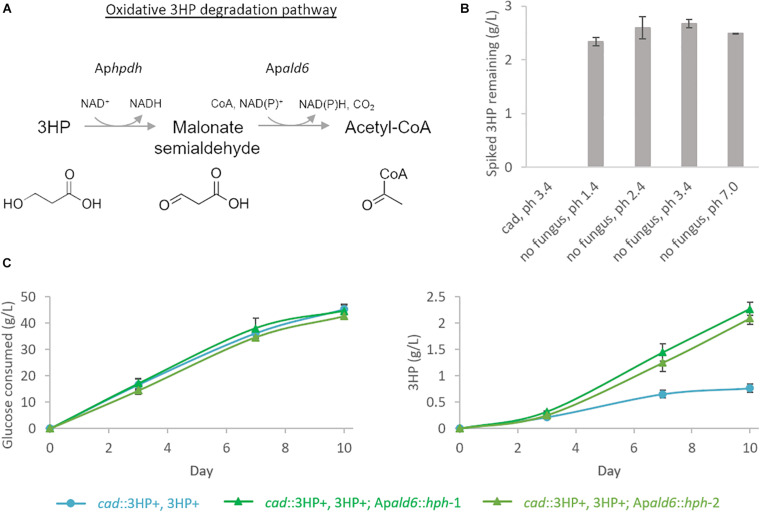
3HP is degraded biotically through Acetyl-CoA by *A. pseudoterreus*. **(A)** Model for 3HP oxidative degradation pathway present in *A. pseudoterreus*. **(B)** Shake-flasks were spiked with 3HP and incubated for 5 days at 30°C with or without the presence of a strain not engineered for 3HP production (*cad*). Uninoculated flasks were examined across a pH range expected to be experienced during *A. pseudoterreus* growth. The expected abiotic degradation product, acrylic acid was not detected in any flask. 3HP was not detected after 5 days incubation with *A. pseudoterreus* (*cad*). **(C)** Deletion of the putative malonate semialdehyde dehydrogenase (Ap*ald6*) improves yield of 3HP from glucose. Two independent deletion mutants are shown.

Production of extracellular 3HP in bioreactor conditions reached a maximum titer of 0.69 g/L, considerably less than in shake-flasks, at hour 67 and then declined steadily to a final titer of 0.17 g/L. Follow-up experiments where exogenous 3HP was incubated in comparable conditions without the presence of a fungus or with an *A. pseudoterreus* strain not engineered for 3HP production demonstrated that 3HP is stable in acidic culture conditions and degraded biotically (Figure 4B). Production of 3HP began tapering off at the on-set of organic acid production suggesting the pathway involved in 3HP degradation is activated during the transition from growth to organic acid production stage.

We examined these results further within the context of metabolic model iJB1325, constructed for the related fungus *Aspergillus niger* (Brandl et al., 2018) and modified by the addition of reactions encoding beta-alanine pyruvate transaminase (beta-alanine + pyruvate ↔ malonate semialdehyde + alanine), 3-hydroxypropionic acid dehydrogenase (malonate semialdehyde + NADPH ↔ 3-hydroxypropionic acid + NADP+), and associated transport reactions and drains. Consumption and production rates for glucose, CO_2_, 3HP, aconitic acid, isocitric acid, citric acid, alpha-ketoglutaric acid, malic acid, and glycerol were used to constrain the model for pFBA. We found that the model is able to recapitulate the slow growth of *A. pseudoterreus* observed in the bioreactor but that all consumed 3HP is secreted as beta-alanine regardless of objective function. Within the metabolic network, 3HP can be reversibly metabolized to beta-alanine, but due to irreversibility of aspartate 1-decarboxylase (aspartate → beta-alanine + CO_2_) and the energy requirement to metabolize excess beta-alanine through the pantothenate biosynthetic pathway, it must be secreted under steady-state. However, extracellular beta-alanine was not observed in shake-flask or bioreactor cultures. This suggests that existing pathway(s) to metabolize 3HP are not represented within the metabolic model.

In bacteria pathways have been identified to directly metabolize 3HP via a reductive route to propionyl-CoA (Schneider et al., 2012) or an oxidative route to malonate semialdehyde, essentially the reverse of the final reaction step of the metabolic pathway deployed here (Zhou et al., 2014). Malonate semialdehyde is then degraded to acetyl-CoA by malonate semialdehyde dehydrogenase (Talfournier et al., 2011). In the fungus *Candida albicans*, 3HP is degraded by the oxidative pathway described above by the enzymes Hpdhp and Ald6p (Otzen et al., 2014). Genes encoding enzymes homologous to the oxidative 3HP degradation pathway were identified by BlastP in *A. pseudoterreus*. The best BlastP hit for Ald6p (*E*-value 2E-114) is among the most significantly up-regulated genes during production phase (*p* < 1E-7 from days 4 to 8) in shake-flasks in strains engineered for 3HP production while the best BlastP hit for Hpdhp (*E*-value 8E-34) was not detected by global proteomics.

It is expected that both the native 3HP dehydrogenase (Ap*hpd1*) and the overexpressed version from *E. coli* (*hpdh*) are reversible and it is therefore unlikely that we could eliminate 3HP degradation while retaining the engineered pathway. We therefore focused on elimination of the competing degradation pathway at the point of malonate semialdehyde by deleting the putative malonate semialdehyde dehydrogenase (Ap*ald6*). In shake-flasks the yield of 3HP is improved by 3.4× in the Ap*ald6* mutant (Figure 4C) confirming the presence and impact of this competing pathway in *A. pseudoterreus*.

**FIGURE 5:**
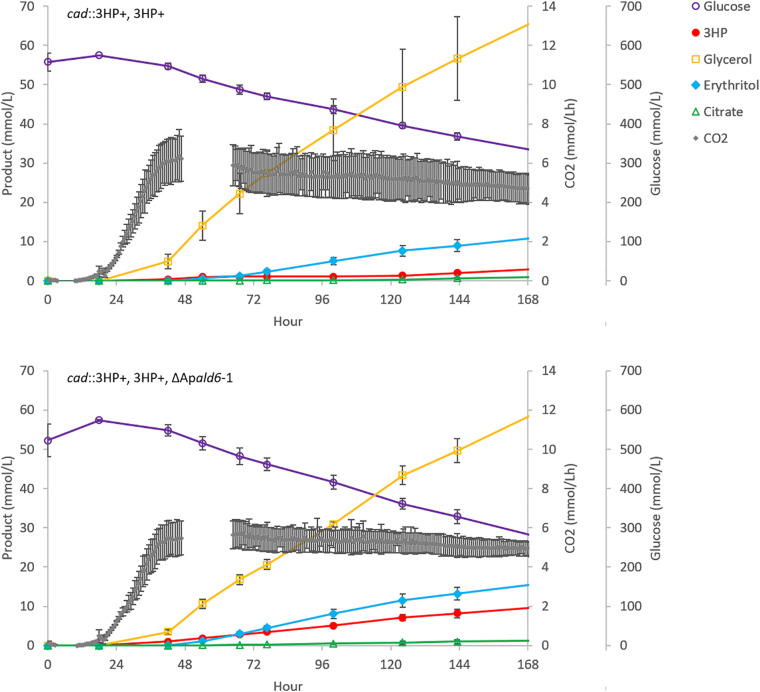
Deletion of A*pald6* increases 3HP yield at 0.5 L bioreactor scale. Time-course production of 3HP and other detected bioproducts. Error bars represent the standard deviation from three replicates.

The ΔAp*ald6* and parent strains were then scaled-up to 0.5 L in bioreactors to re-evaluate 3HP production with pH control and improved aeration (Figure 5). By day seven the parent strain had accumulated 0.27 ± 0.10 g/L 3HP while yield was improved by 3.3 × to 0.88 ± 0.11 g/L 3HP in the Ap*ald6* mutant. We did not observe actively decreasing 3HP in shake-flask or small-scale bioreactor experiments as we did in the 20 L bioreactor, but improved yield suggests that flux toward acetyl-CoA is a major competitor with flux toward 3HP from malonate semialdehyde. At 0.5 L scale the rate of CO_2_ evolution was slower while higher levels of glycerol and erythritol were accumulated. The major product during cultivation in the 20L bioreactor, aconitic acid, was not observed in the 0.5 L bioreactors supporting the hypothesis that a shift to organic acid production coincides with loss of 3HP. The predicted reaction catalyzed by malonate semialdehyde dehydrogenase (EC 1.2.1.27; Figure 4A) was added to the metabolic model. This modification eliminates production of unobserved metabolites during pFBA and improves the consistency of the model with empirical observations from bioreactor and shake-flask cultivations.

## Discussion

Biological engineering of microorganisms is a promising route to sustainable manufacturing of fuels and chemicals that are currently derived from the petroleum industry. Here we focused on engineering an acidophilic filamentous fungal host, *A. pseudoterreus*, for conversion of sugars to the polymer precursor 3HP. Production of 3HP using *A. pseudoterreus* is possible in acidic conditions at a pH of 2–3 where 3HP is primarily in the protonated free-acid form, potentially reducing the cost of downstream separations. Overexpression of a synthetic pathway that has been shown to function in yeast (Borodina et al., 2015) demonstrated that production of 3HP is feasible using *A. pseudoterreus* but initial yield was poor. Understanding low productivity is challenging in non-model systems where limited information is available regarding metabolic flux and its control. Here we applied multi-omic analyses of engineered *A. pseudoterreus* strains to generate a systems-level snapshot of metabolites and proteins involved in 3HP production and metabolism as a whole. This approach quantified the effect of initial and increased expression level of the 3HP pathway on the bioconversion host and identified pathways competing with 3HP production.

Establishment of the beta-alanine pathway for 3HP biosynthesis had a greater overall effect on metabolism than increased expression level of the pathway and resulted in substantially increased intracellular pools of beta-alanine, 2,4-diaminobutanoate, and 3HP and up-regulation of malonate-semialdehyde dehydrogenase (Ap*ald6*), enzymes involved in 4-aminobutanoate (GABA) degradation to succinate (Ap*uga1*, Ap*uga2*), and genes that may be involved in metabolism of 3HP pathway intermediates through a CoA bound intermediate (Ap*suc5*, succinyl-CoA:3-ketoacid-CoA transferase; Ap*adh11*, hydroxyacid-oxoacid transhydrogenase; Ap*coaT*, acyl-CoA:carboxylate-CoA transferase; Ap*ahd2*, aldehyde dehydrogenase; Ap*acat1*, keto-acyl-CoA thiolase; and Ap*acat2*, acyl-CoA thiolase), similar to the 3HP/4HB CO_2_ fixation cycle described in archaea (Berg et al., 2007).

The GABA degradation response to the 3HP pathway ties nitrogen and carbon metabolism together through succinate. In yeast, *uga1* and *uga2* are up-regulated in response to the presence of GABA (Ramos et al., 1985), however we did not detect a significant impact on the intracellular GABA pool size in response to the presence of the 3HP pathway. 4-hydroxybutanoate (GHB) was also accumulated in the engineered strains (Supplementary File 1). In yeast, there is evidence to support the presence of a succinate semialdehyde dehydrogenase to produce GHB (Bach et al., 2009) but not for the reverse reaction catalyzed by GHB dehydrogenase (Mekonnen and Ludewig, 2016), though *Aspergillus* species with a larger genome may catalyze this reaction. Elucidation of the genes responsible in fungi would aid in understanding the effect of 3HP on GABA and GHB metabolism. It is likely that accumulation of 2,4-diaminobutanoate is a direct result of increased Ap*uga1* expression (Figure 2), with the proposed precursor aspartate semialdehyde being supplied by an enhanced aspartate pool through the activity of aspartate kinase and aspartate semialdehyde dehydrogenase, though neither enzyme is significantly differentially expressed in response to the presence of the 3HP pathway (Supplementary File 1).

Extracellular metabolomics during bioreactor cultivation revealed that 3HP is produced and then metabolized at the onset of organic acid production. This led us to search for metabolic pathways involved in catabolism of 3HP, which are poorly characterized in fungi. During shake-flask cultivation the methylmalonate-semialdehyde dehydrogenase (g153; Ap*ald6*) is strongly up-regulated at all times in the engineered strains. This enzyme, normally involved in branched-chain amino acid degradation, has been implicated in 3HP metabolism in bacteria (Zhou et al., 2014; Arenas-Lopez et al., 2019) and the yeast *C. albicans* (Otzen et al., 2014) by also acting as a malonate-semialdehyde dehydrogenase and diverting flux toward acetyl-CoA rather than 3HP. Here we deleted a putative malonate-semialdehyde dehydrogenase Ap*ald6* and found that 3HP yield was drastically improved (Figure 4). Reductive degradation of 3HP via propanoyl-CoA synthase has also been demonstrated in bacteria (Schneider et al., 2012), and while homologs of the enzymes involved are not readily identifiable in fungi, we have identified a variety of significantly up-regulated enzymes that may be directly involved in alternative 3HP assimilation pathways. Further genetic characterization will reveal the metabolic repertoire that fungi express to metabolize 3HP.

Multi-omic technologies offer a promising route to systematic analysis of bioconversion processes and identification of targets for improvement of metabolic flux. The results in this study demonstrate that 3HP production can be effectively improved by application of multi-omic analyses to further our system level understanding of genetically engineered *A. pseudoterreus*. However, extracellular metabolomics in shake-flasks and bioreactor scale-up revealed a wide variety of co-products being produced at levels higher than that of 3HP. These co-products compete metabolically with production of 3HP and contaminate the desired end-product, making separations impractical. Improvement of 3HP production in *A. pseudoterreus* will require minimization of these co-products either through growth optimization or further metabolic engineering.

## Data Availability Statement

The raw data supporting the conclusions of this article will be made available by the authors, without undue reservation.

## Author Contributions

KP, ZD, NM, Y-MK, YG, SD, JK, BH, JC, EP, B-JW-R, JZ, HD, SB, KB-J, NH, and JM conceived and designed the experiments. KP, ZD, NM, Y-MK, YG, SD, JK, BH, MS, TL, JC, EP, B-JW-R, CN, and KB-J performed the experiments and analyzed the data. KP, ZD, NM, Y-MK, YG, and JM wrote the manuscript. All authors read and approved the final manuscript.

## Conflict of Interest

The authors declare that the research was conducted in the absence of any commercial or financial relationships that could be construed as a potential conflict of interest.

## Footnotes

1 https://agilebiofoundry.org/

2 https://omics.pnl.gov/software/masic-results-merger
